# Health Department Accreditation as a Catalyst to Foster the Development of a Future Public Health Workforce

**DOI:** 10.3389/fpubh.2015.00020

**Published:** 2015-02-02

**Authors:** Hugh H. Tilson, Kaye W. Bender, Jessica L. Kronstadt

**Affiliations:** ^1^University of North Carolina Gillings School of Global Public Health, Chapel Hill, NC, USA; ^2^Public Health Accreditation Board, Alexandria, VA, USA

**Keywords:** public health accreditation, workforce, undergraduate education, health department, training support

The relatively new Public Health Accreditation Board (PHAB) proffers a new force to bring attention to the need for undergraduate, graduate, and continuing education for those working in public health. The workforce of federal, state, and local health departments in the United States comprises more than 450,000 individuals representing a wide variety of disciplines, from nurses to sanitary engineers to health educators (1). Despite the many professionals with graduate training in clinical and other disciplines, the majority of those working in local public health departments have baccalaureate-level or less education (2) – making undergraduate college a key focus for public health workforce training. A projected shortage in the future raises concerns about the capacity and capabilities of those working to promote public health (2). This Opinion reveals how PHAB standards reinforce the need to ensure the pipeline for trained public health department workers. In addition, early evidence suggests that accredited health departments have the capacity to partner with schools to strengthen the workforce. As more health departments pursue accreditation, it will likely provide opportunities to foster partnerships between public health agencies and undergraduate institutions that are preparing individuals to address the practical needs of twenty-first century public health.

## Background

The 2003 Institute of Medicine report entitled “*Who Will Keep the Public Healthy*?” contained a recommendation that “…all undergraduates should have access to education in public health” (1). In the intervening years, great progress has been made by the public health academic community in designing creative approaches at the associate, undergraduate, and graduate degree levels in public health, including the addition of more creative modes of the delivery of public health education, such as just-in-time learning; online learning; and flipped classroom. Also reflecting the importance of providing opportunities for students to learn about public health, Healthy People 2020 includes several objectives related to increasing the proportion of schools that offer public health majors, minors, associate degrees, and certificate programs. For example, Objective PH-4.1 seeks to “increase the proportion of 4-year colleges and universities that offer public health or related majors” from a baseline of 7% in 2008 to 10% in 2020 (3). A recent article by Drehobl et al. described the current challenges and solutions facing the public health workforce and summed up the issue in one, salient phrase “Lack of the right number of people with the right skills in the right place at the right time” and described various means by which the public health workforce could be made stronger (4).

A parallel and complementary body of work that occurred in the same decade was the launch of the national voluntary accreditation program that paved the way for public health department accreditation to become a reality. A formal process to explore the feasibility of accreditation (5) was sparked by a recommendation in another 2003 Institute of Medicine report, *The Future of Public Health in the 21^st^ Century*, which noted that accreditation might be a mechanism to strengthen performance and accountability for governmental public health departments (6). The PHAB launched the national voluntary accreditation program in September 2011, after several years of development and the input from many individuals and organizations (7). As of December 2014, almost 280 of the nation’s approximately 2,400 local health departments, as well as 28 state and 2 tribal health departments are actively pursuing accreditation, including 60 that have already achieved that milestone (8).

The PHAB accreditation standards and measures are based on a public health framework of the three core functions of public health and the 10 Essential Public Health Services (9). Version 1.5 of the PHAB Standards and Measures became effective July 1, 2014, with the intention of clarifying or strengthening the initial version of the accreditation standards and measures. Attention to the public health workforce is a key area within the accreditation standards and received special attention in the July 2014 revisions (10).

Each standard has one or more corresponding measures. (See http://www.phaboard.org/accreditation-process/public-health-department-standards-and-measures/ for the full text of the standards and measures.) Applicant health departments are reviewed by a team of peers who assesses conformity with each of the measures and includes their findings in a Site Visit Report. The PHAB Accreditation Committee, a majority of whose members have recent or current experience in governmental public health practice, review the report, and make the accreditation determination. The reports of the first 62 health departments to be reviewed by the Accreditation Committee provide insights on how well the health departments that were among the first to complete the PHAB application process performed on the workforce measures. Some of these insights are shared below.

## Health Department Accreditation and Workforce Development

The national accreditation standards and measures contain several expectations about workforce development, with two key components. One area of focus is the health department’s role in developing the pipeline of future public health workers (Standard 8.1: Encourage the Development of a Sufficient Number of Qualified Public Health Workers). The second is health departments’ responsibility for recruiting, hiring, and developing their workforce (Standard 8.2: Ensure a competent workforce through assessment of staff competencies, the provision of individual training and professional development, and the provision of a supportive work environment) (9).

From PHAB’s perspective, maintaining a competent public health workforce requires a supply of qualified public health workers in sufficient numbers to meet public health needs. As public health workers retire or seek other employment opportunities, it is essential that newly trained public health workers enter the field. Trained and competent workers are needed in such diverse areas as epidemiology, health education, community health, public health laboratory science, public health nursing, environmental public health, and public health administration and management. Every health department has a responsibility to collaborate with others to encourage the development of a sufficient number of public health students and to encourage qualified individuals to enter the field in order to meet the staffing needs of public health departments and other public health organizations.

Measure 8.1.1 is designed to assess the health department’s activities, initiatives, and strategies aimed at encouraging public health as a career choice. PHAB expects that health departments will work with schools, academic programs, or other organizations as a means to promote public health as a viable career choice. Health departments can document a variety of examples of partnerships in this area, including collaboration with a school or college of public health; working with organizations such as AmeriCorps; coordinating with a high school to make presentations to students about public health careers; working with vocational training schools to promote public health; partnering with a 4H club to provide information about public health to members; guest lecturing at a community college; or providing after school observation experiences for high school or undergraduate students. Examples of more robust academic involvement include: student placements or practicums; academic service learning; internship opportunities; faculty positions or guest lectures by health department staff; participation in high school, university, college, or Tribal college programs; and/or job/career fairs. Collaborations to build the pipeline is an area in which the first set of health departments to be reviewed by the Accreditation Committee excel, with more than 90% fully demonstrating their conformity with Measure 8.1.1. Based on PHAB’s experiences to date, fostering the development of future public health workers remains a priority for health departments seeking to obtain and maintain accreditation.

However, there are still opportunities for improvement in the other workforce measures, as only approximately 60% fully demonstrated conformity with Measure 8.2.1 (Maintain, implement, and assess the health department workforce development plan that addresses the training needs of the staff and the development of core competencies). The IOM workforce report called upon schools of public health to “fulfill their responsibility for assuring access to life-long learning opportunities for public health professionals, other members of the public health workforce, and other health professionals who participate in public health activities” (1). Health departments can work with all types of colleges and universities to develop programs to effectively meet health departmental training needs for improving employees’ job performance in support of the population’s health (2).

Health departments often struggle with recruiting qualified public health workers who reflect the diversity of the population of the health department’s jurisdiction. For this reason, a new requirement has been added to Version 1.5 of the Standard and Measures explicitly requiring the “recruitment of individuals who reflect the population served” (Measure 8.2.2) (9). Multiple creative strategies are needed to assist health departments in their ongoing desire to recruit and hire the most well prepared and diverse workforce possible – and collaborating with local college programs, particularly ones that enroll a diverse student body, can be one such strategy. As an example of this type of partnership, one accredited health department collaborated with a local academic institution on a summer program to help spread awareness about public health among high school students living in the urban core.

## Conclusion

Preparation for accreditation may motivate health departments to reach out to schools in their communities. Similarly, schools may use the accreditation standards as guidance, as they consider ways to better engage their colleagues in public health practice. As health departments fulfill the expectations for Standard 8.1, they will likely strengthen or build new relationships with undergraduate educational institutions and others. These partnerships, in turn, can help the departments to achieve Standard 8.2 by providing high quality, locally accessible opportunities for continued development of competencies, and life-long learning.

Excellence in public health practice requires a commitment to fostering the development of a strong public health workforce. As public health departments continue to maintain and achieve accreditation, an ongoing partnership with educational programs for public health workforce development will be essential. The collaborations between academic programs in public health and progressive state, local, tribal, and territorial health departments are already paving the way for a stronger collaboration for the future. Accreditation helps shine the spotlight on these critical partnerships.

## Conflict of Interest Statement

Jessica L. Kronstadt and Kaye W. Bender are employees of the Public Health Accreditation Board. Hugh H. Tilson is a volunteer member of the Board.
